# Effect of Early Extracorporeal Shockwave Therapy on Postoperative Pain and Functional Recovery After Intramedullary Nailing: An Open-Label Randomized Controlled Trial

**DOI:** 10.3390/life15111704

**Published:** 2025-11-03

**Authors:** Yonghyun Yoon, Jihyo Hwang, Jaeyoung Lee, King Hei Stanley Lam, Jeimylo C. de Castro, Hyeongjik Kim, Dongyeun Sung, Seungbeom Kim, MinJae Lee, Chanwool Park

**Affiliations:** 1 Department of Orthopaedic Surgery, Gangnam Sacred Heart Hospital, Hallym University College of Medicine, 1 Singil-ro, Yeongdeungpo-gu, Seoul 07441, Republic of Korea; 2 Incheon Terminal Orthopedic Surgery Clinic, Inha-ro 489beon-gil, Namdong-gu, Incheon 21574, Republic of Korea; 3 International Academy of Regenerative Medicine, Inha-ro 489beon-gil, Namdong-gu, Incheon 21574, Republic of Korea; 4 International Academy of Musculoskeletal Medicine, MSKUS, 1035 E. Vista Way #128, Vista, CA 92084, USA; 5 Faculty of Medicine, The University of Hong Kong, Hong Kong; 6 Faculty of Medicine, The Chinese University of Hong Kong, New Territory, Hong Kong; 7 The Board of Clinical Research, The International Association of Musculoskeletal Medicine, Kowloon, Hong Kong; 8 SMARTMD Center for Non-Surgical Pain Interventions, Makati 1205, Philippines; 9 Research Department, Adventist University of the Philippine (A.U.P.), Silang Cavite 4118, Philippines; 10 Ahyeon Orthopedic Clinic, B270 Sinchon-ro, Mapo-gu, Seoul 04116, Republic of Korea; 11 Himchannamu Neurosurgery Clinic, 439, Hakjeong-ro, Buk-gu, Daegu 41423, Republic of Korea; 12 Miso Pain Clinic, 1569, Bongyeong-ro, Yeongtong-gu, Suwon-si 16703, Republic of Korea

**Keywords:** extracorporeal shockwave therapy, intramedullary nailing, hip fracture, postoperative pain, randomized controlled trial, functional recovery

## Abstract

Background/Objectives: Intramedullary (IM) nailing for hip fractures can cause iatrogenic abductor muscle injury, leading to pain and functional impairment. This study evaluated whether early extracorporeal shockwave therapy (ESWT) safely accelerates recovery. Methods: In this open-label randomized controlled trial, 51 patients (≥50 years; intention-to-treat: ESWT *n* = 26; control *n* = 25) received either standard postoperative care (control) or standard care plus three ESWT sessions. The primary outcome was pain (Visual Analog Scale, VAS); the secondary outcome was hip function (modified Harris Hip Score, mHHS), assessed at 3, 6, and 12 months. Results: Linear mixed-effects modeling showed significantly faster pain reduction in the ESWT group (group × time β = 0.086 points/month; *p* = 0.027), corresponding to an additional 1.0-point VAS reduction over 12 months. Functional improvement (mHHS) did not reach statistical significance (group × time β = 0.485; *p* = 0.462). No ESWT-related adverse events were observed. Conclusions: Early postoperative ESWT is a safe adjunctive therapy that accelerates pain relief after IM nailing for hip fractures. Although functional improvements were not statistically significant, pain reduction may facilitate early mobilization and rehabilitation.

## 1. Introduction

Hip fractures in elderly patients carry substantial morbidity and mortality, with rates remaining significant despite modern surgical care. Intramedullary (IM) nailing has become the standard surgical treatment for proximal femoral fractures due to its biomechanical stability and facilitation of early weight-bearing [1,2,3].

However, a clinically important but often underappreciated complication of IM nailing is iatrogenic injury to the hip abductor muscles. The nail’s entry point through the greater trochanter places the gluteus medius and minimus tendinous insertions at direct risk [4,5,6,7]. Abductor muscle injury is highly prevalent, occurring in approximately 30–45% of IM nailing cases, with sonographic studies reporting rates as high as 57% when examined postoperatively. These injuries result in persistent pain, debilitating Trendelenburg gait, and impaired balance, significantly delaying functional recovery [8,9].

Current management of postoperative abductor injuries relies primarily on analgesics and physical therapy, which are often insufficient to manage acute pain in elderly patients. This creates a critical clinical gap for interventions that can safely accelerate tissue healing without compromising outcomes in this vulnerable population. Extracorporeal shockwave therapy (ESWT) has emerged as a promising non-invasive modality for promoting tissue healing.

ESWT promotes tissue regeneration through mechanotransduction, converting mechanical stimuli into cellular healing responses via angiogenesis and growth factor expression [10,11]. While efficacy is well-established for chronic tendinopathies such as greater trochanteric pain syndrome [12,13,14,15,16], its use for acute, postoperatively induced muscle injuries in elderly patients remains unexplored. Given that faster pain relief could reduce opioid consumption, facilitate early mobilization, and prevent immobility-related complications, investigating ESWT in this setting is clinically important.

This study evaluated whether early postoperative ESWT accelerates pain reduction and improves functional outcomes in elderly patients with abductor injury following IM nailing for hip fractures.

## 2. Materials and Methods

### 2.1. Study Design and Ethical Approval

This prospective, single-center, randomized controlled trial was conducted at the Department of Orthopedic Surgery, Hallym University Kangnam Sacred Heart Hospital, between May 2023 and December 2024. The study protocol was approved by the Institutional Review Board (IRB) of Hallym University Kangnam Sacred Heart Hospital (No. 2022-12-004-007, approved: 2 May 2023) and adhered to the principles of the Declaration of Helsinki. Written informed consent was obtained from all participants.

The trial was registered retrospectively (Clinical Research Information Service, CRIS No. PRE20250926-004) due to administrative delays in the registration process at the time of patient enrollment. This retrospective registration may introduce potential reporting bias; however, this risk is mitigated by: (1) the study protocol and primary outcomes were established prior to patient enrollment; (2) outcome assessments were conducted by personnel blinded to group allocation; and (3) no outcome measures were changed after trial commencement. All pre-specified outcomes (VAS and mHHS) were consistently measured at all scheduled time points.

### 2.2. Participants

Inclusion Criteria:

- Age ≥ 50 years;
- Unilateral proximal femoral fracture treated with IM nailing;
- Able to provide informed consent.

Exclusion Criteria:

- Non-ambulatory status preoperatively (Koval Grade 6);
- Cognitive impairment preventing informed consent or outcome assessment;
- Active infection at the surgical site at the time of enrollment;
- Severe cardiac arrhythmia or hemodynamic instability requiring ICU care;
- Pregnancy or refusal to participate;
- Prior ESWT within 3 months;
- For the ESWT group specifically: inability to complete all three scheduled ESWT sessions.

Fracture Classification: Fracture type (intertrochanteric, subtrochanteric, femoral shaft) was recorded for descriptive purposes but was not used as a stratification variable in randomization. Preliminary analysis showed no significant association between fracture type and pain outcomes, so laterality (left vs. right) was selected as the sensitivity analysis variable to account for the observed baseline imbalance.

**Sample size.** We calculated the required sample size a priori for the primary outcome (pain VAS trajectory at 12 months). Assuming a between-group mean difference (MCID) of 2.0 cm on a 10 cm VAS, a common SD of 2.6 cm, two-sided α = 0.05 and 80% power, the required sample size was 26 participants per group using a two-sample comparison. Allowing for 10% attrition, the target enrollment was 29 per group (total 58). Each arm ceased enrollment upon reaching its target.

**Figure 1:**
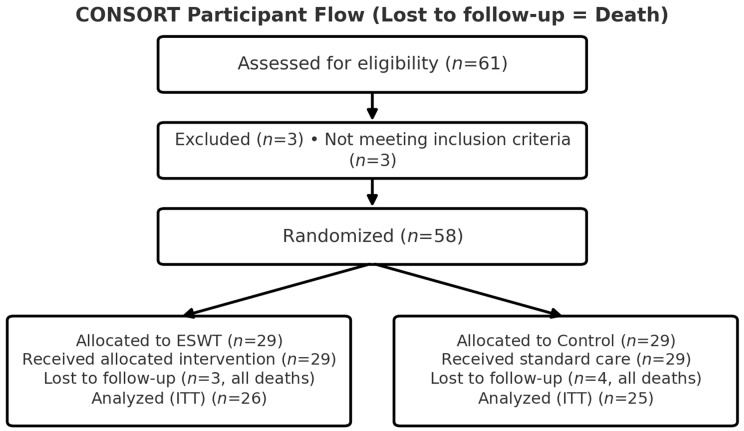
CONSORT participant flow diagram. Assessed for eligibility (*n* = 61); excluded (*n* = 3) for not meeting inclusion criteria; randomized (*n* = 58). Allocated to ESWT (*n* = 29): received intervention (*n* = 29); lost to follow-up (*n* = 3, all deaths due to medical comorbidities); analyzed intention-to-treat (*n* = 26). Allocated to Control (*n* = 29): received standard care (*n* = 29); lost to follow-up (*n* = 4, all deaths due to medical comorbidities); analyzed intention-to-treat (*n* = 25).

Initially, 61 patients were assessed for eligibility; 3 were excluded (not meeting inclusion criteria). 58 were randomized 1:1 (ESWT *n* = 29, Control *n* = 29). During follow-up, 3 participants in the ESWT group and 4 in the Control group died, yielding 51 patients for the intention-to-treat analysis (ESWT *n* = 26, Control *n* = 25; Figure 1).

### 2.3. Randomization and Blinding

**Randomization**. Participants were randomized 1:1 using a computer-generated permuted block design with a fixed block size of 4; no stratification factors were used. Due to the nature of the intervention, the surgeon and the ESWT provider were not blinded. However, the dedicated clinical researcher who assessed all patient outcomes was blinded to group allocation to minimize detection bias.

**Allocation concealment**. Allocation was concealed using sequentially numbered, opaque, sealed envelopes (SNOSE) prepared by an independent coordinator not involved in enrollment or assessment. Envelopes were tamper-evident and were opened after baseline assessment to reveal the assignment. **Implementation**. An independent statistician generated the random sequence; a research nurse enrolled participants; and a therapist not involved in outcome assessment assigned the interventions.

### 2.4. Interventions

Both groups received identical standard postoperative care, which included a structured rehabilitation program and pharmacologic therapy. The intervention group received three additional ESWT sessions.

#### 2.4.1. Standard Postoperative Care (Control Group)

Standard care was multifaceted, beginning on the first postoperative day. The rehabilitation program included daily sessions of guided exercises, such as straight leg raises, ankle pumps, and gentle hip abduction against gravity, with a goal of 10–15 repetitions per set. Gait training with a walker was initiated as soon as tolerated. Pharmacologic therapy was standardized to include celecoxib for pain management unless contraindicated, low-molecular-weight heparin (LMWH) for DVT prophylaxis for two weeks, and intravenous iron for postoperative anemia when hemoglobin levels fell below 9 g/dL.

#### 2.4.2. ESWT Protocol (Intervention Group)

Extracorporeal shockwave therapy (ESWT) was applied three times postoperatively—on postoperative day 3, day 7, and day 11. Before treatment, patients were informed about the procedure and potential side effects of ESWT. The treatment was performed with the patient positioned in a lateral decubitus posture on the non-affected side, as described in our previous ultrasonography study, which was used to identify abductor muscle injury.

**Figure 2:**
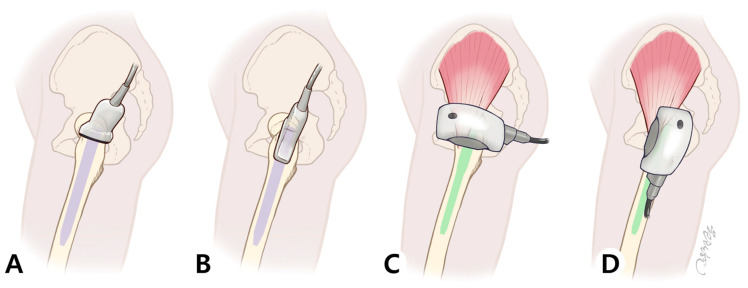
Ultrasound probe and shock wave hand piece position (**A**) Confirmation of the short axis of the greater trochanter facet using ultrasound. (**B**) Confirmation of the long axis of the greater trochanter facet using ultrasound. (**C**) Applying a linear hand piece to the greater trochanter facet along the short axis, as in the ultrasound position. (**D**) Applying a linear hand piece to the greater trochanter facet along the long axis, as in the ultrasound position.

ESWT was administered using the PiezoWave^2^ FBL10x5G2 system (Richard Wolf GmbH, Knittlingen, Germany; distributed by ELvation, Germany) with the following settings: frequency of 6 Hz, energy flux density of 0.16 mJ/mm^2^, penetration depth of 20 mm, and 2000 impulses per session. Crucially, the penetration depth was specifically set to 20 mm to target the injured gluteal tendons and surrounding soft tissues while ensuring the shockwave energy did not reach the surface of the underlying intramedullary nail, thereby preventing any potential adverse effects on the implant. These settings are consistent with established protocols for treating tendinopathies and were chosen to maximize therapeutic benefit while ensuring patient safety [17]. A layer of sterile chlorhexidine gel was applied to the greater trochanter (GT) area—near the surgical incision—before placing the applicator head to ensure precise delivery of energy to the GT fossa (Figure 2).

To perform an ultrasound, the patient was first positioned in the lateral decubitus position. The hip on the side to be examined was flexed to 15–30°, and the knee was flexed to 30°. (A) In the short-axis (SAX) view, the transducer was placed on the apex of the GT in a transverse plane perpendicular to the femoral shaft. (B) In the long-axis (LAX) view, the transducer was placed on the apex of the GT in a plane parallel to the femoral shaft. (C,D) ESWT was applied in the same patient position as the ultrasound examination. The applicator was placed over the GT region to deliver energy precisely to the GT fossa, which corresponds to the entry point of the nail and the site where abductor injury typically occurs.

### 2.5. Outcome Measures

The primary outcome was pain intensity, assessed using the Visual Analog Scale (VAS). The VAS is a 10 cm line where 0 represents “no pain” and 10 represents the “worst imaginable pain.”

The secondary outcome was functional recovery, evaluated using the modified Harris Hip Score (mHHS). The mHHS is a patient-reported outcome measure assessing pain and function.

All outcomes were measured at scheduled outpatient follow-up visits at 3, 6, and 12 months postoperatively.

### 2.6. Statistical Analysis

All analyses were performed in R 4.4.2 using an intention-to-treat dataset (*n* = 51: 25 control, 26 ESWT).

Primary Analysis

Baseline characteristics were compared between groups using independent samples *t*-tests (continuous variables) or chi-square/Fisher’s exact tests (categorical variables). Results are reported as mean ± SD. Longitudinal changes in pain (VAS) and functional outcomes (mHHS) were modeled using linear mixed-effects regression with restricted maximum likelihood estimation. The primary model included fixed effects for time (months), treatment group, and their interaction. All models included a random intercept for each participant to account for repeated measurements within individuals. Model parameters were estimated by restricted maximum likelihood; *p*-values and 95% confidence intervals were derived using Satterthwaite’s approximation.

Baseline Imbalance and Sensitivity Analysis

A chi-square test revealed significant baseline imbalance in fracture laterality between groups (left-sided: 40.0% control vs. 86.2% ESWT; χ^2^ = 8.756, *p* = 0.003). To assess whether this imbalance confounded the primary findings, sensitivity analysis was performed by including laterality as a fixed covariate in the mixed-effects model. Stratified analyses were also conducted separately for left-sided and right-sided fractures to evaluate treatment effect consistency across laterality subgroups. Results of sensitivity and stratified analyses are detailed in Supplementary Tables S1 and S2.

Clinical Significance

Clinical relevance was assessed by calculating the proportion of participants achieving minimal clinically important difference (MCID) for the mHHS (≥8-point improvement) across three intervals: 3–6 months, 6–12 months, and 3–12 months. Group differences were tested using chi-square tests. Adjusted means for each follow-up time point were derived from the mixed models; Welch *t*-tests compared baseline-to-follow-up VAS reductions between groups.

Model Assumptions and Missing Data

Normality of residuals was assessed graphically (Q-Q plots) and formally (Shapiro–Wilk test). Homoscedasticity and independence of residuals were evaluated graphically. Given the robustness of linear mixed-effects models to moderate violations of normality in our data, no transformations were applied. The sphericity assumption is not applicable to mixed-effects models. Seven patients died during follow-up (3 ESWT, 4 control) at various time points (range: 2–11 months post-surgery) due to medical comorbidities unrelated to intervention (cardiac failure *n* = 3, respiratory failure *n* = 2, sepsis *n* = 1, stroke *n* = 1). Mortality did not differ between groups (*p* = 1.000, Fisher’s exact test). For patients who died, data were included up to their last assessment and treated as censored observations in longitudinal analyses (i.e., excluded from subsequent time points after death) rather than completely removed from the dataset. Maximum-likelihood estimation inherently handles missing data (<5%) without imputation. The study protocol and statistical analysis plan are available from the corresponding author upon request.

## 3. Results

### 3.1. Participant Flow and Baseline Characteristics

Of the 61 patients screened, 58 were randomized (ESWT *n* = 29, Control *n* = 29). During follow-up, 7 deaths occurred (3 in ESWT; 4 in Control), resulting in ESWT *n* = 26 and Control *n* = 25 for the intention-to-treat analysis (Figure 1).

The mean age of the participants was 80.5 ± 8.3 years, and 78.4% were female. There were no statistically significant between-group differences in age or sex distribution. However, there was a significant difference in fracture laterality, with a higher proportion of left-sided fractures in the ESWT group compared to the control group (84.6% vs. 40.0%, *p* = 0.003). Other baseline characteristics, including fracture diagnosis and preoperative Koval grade, were comparable between the groups (Table 1).

### 3.2. Pain Outcomes (VAS)

**Figure 3:**
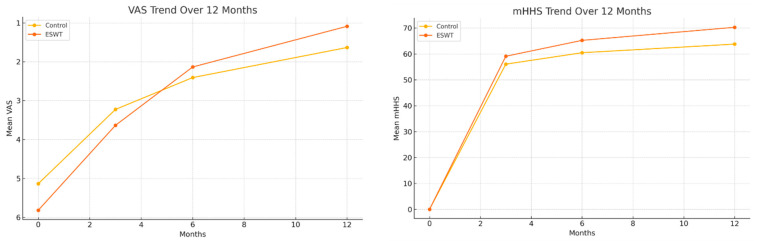
Trends of VAS and mHHS over 12 Months.

Over the 12-month follow-up period, both groups showed a progressive reduction in pain, with detailed scores presented in Table 2 and the overall trend visualized in Figure 3. The linear mixed-effects model revealed that pain scores (VAS) decreased by an average of 0.337 points per month across all participants (*p* < 0.001).

Pain decreased over time; under our coding scheme, a positive month coefficient reflects a downward VAS trajectory (β = +0.337 points/month; *p* < 0.001). Accordingly, the positive group × time interaction (β = +0.086 points/month; *p* = 0.027) indicates a faster VAS decline with ESWT than with control, corresponding to ~1.0 additional VAS-point reduction over 12 months (Table 3).

### 3.3. Sensitivity Analysis: Accounting for Baseline Imbalance

After including laterality as a fixed covariate, the 12-month ESWT effect remained statistically significant (adjusted difference ≈−1.20; *p* ≈ 0.01), whereas the 3- and 6-month effects were not significant (e.g., 6 months *p* ≈ 0.17). These results are consistent with the unadjusted model, indicating that laterality did not materially alter the primary inference (Supplementary Table S1).

In laterality-stratified analyses, the 12-month effect was numerically larger among left-sided fractures (*n* = 32; ESWT 22, Control 10), while no time point reached significance among right-sided fractures (*n* = 19; ESWT 4, Control 15), likely reflecting limited precision (Supplementary Table S2).

### 3.4. Functional Outcomes (mHHS)

Similarly, functional scores (mHHS) improved significantly over time in both groups, with an average increase of 4.732 points per month (*p* < 0.001). The specific mHHS values for each time point are detailed in Table 2, and the trend is shown in Figure 3. Although the ESWT group demonstrated a trend toward greater functional improvement over 12 months, the group × time interaction was not statistically significant (β = +0.485 points/month, *p* = 0.462) (Table 4).

Using an MCID of an 8-point increase in the mHHS [18], responder proportions did not differ significantly between groups; across 3–12 months, the control group showed higher responder rates than ESWT (64.0% vs. 50.0%; Table 5).

### 3.5. Safety and Adverse Events

No immediate or delayed adverse events related to the ESWT application were observed during the study period.

## 4. Discussion

### 4.1. Principal Findings and Deeper Interpretation

**Figure 4:**
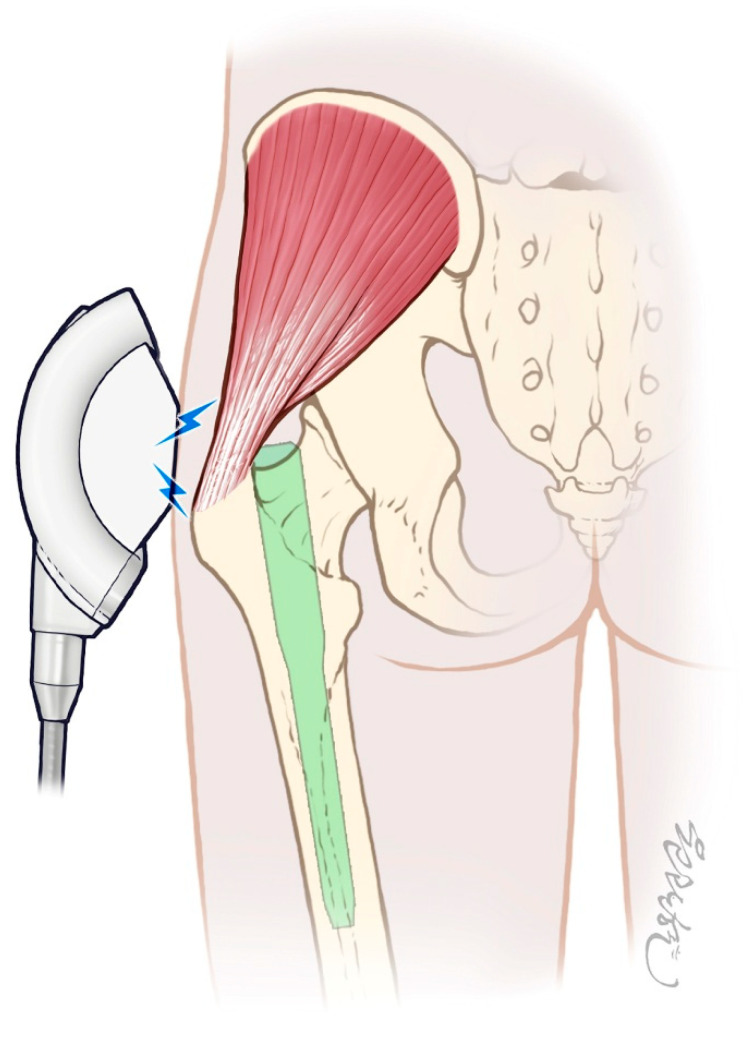
The ESWT was applied while the implant was present, but the depth of the handpiece was set to 20 mm so that the energy was not delivered to the implant, but only to the soft tissue above. The implants marked in green indicate that the shock wave energy does not reach there, and the shock wave energy is indicated by a lightning bolt symbol.

The principal finding of this study is that an early, three-session ESWT protocol demonstrates significant improvement in the trajectory of postoperative pain relief in elderly patients who underwent IM nailing for hip fractures (Figure 4). This is, to our knowledge, the first randomized controlled trial to demonstrate this benefit in an acute, postoperative setting with an internal fixator in place. By setting the penetration depth to 20 mm, we focused the therapeutic energy on the overlying abductor tendons and muscles without contacting the metallic implant, addressing a key safety concern in the acute postoperative setting. In early tissue injury, ESWT can down-regulate pro-inflammatory mediators and substance P; such modulation likely contributed to the faster pain resolution observed in the intervention group. Mild adverse effects (e.g., local soreness or erythema) have been reported infrequently in prior ESWT studies [17].

While statistically significant (*p* = 0.027), the absolute VAS difference of approximately 1.0 point on a 10-point scale over 12 months represents modest clinical benefit, particularly in the context of a limited sample size (*n* = 51) and single-center design.

However, a critical finding was the dissociation between pain improvement and functional gain. The statistically significant pain reduction did not translate into a correspondingly significant improvement in functional scores (mHHS). This suggests that while ESWT effectively addresses the nociceptive and inflammatory aspects of the injury, functional recovery is a more complex, multifactorial process. The initial trauma and surgery result in substantial muscle atrophy, impaired neuromuscular control, and kinesiophobia (fear of movement), which are not directly targeted by ESWT. Therefore, while ESWT can create a more favorable, low-pain environment for recovery, it is not a substitute for the targeted, progressive resistance training and neuromuscular re-education required to restore abduction strength and gait mechanics. The control group’s natural recovery pattern mirrored prior reports of gradual pain reduction [18].

Furthermore, the choice of the mHHS as the primary functional outcome warrants critical reflection. The mHHS is a valuable patient-reported outcome measure, but as a composite score, it may lack the sensitivity to detect subtle but clinically important changes in the early postoperative period. Future studies could benefit from incorporating more granular, performance-based measures such as gait analysis to objectively quantify Trendelenburg limp, dynamometry to directly measure abductor strength, or timed up-and-go (TUG) tests to assess dynamic balance and mobility. Such objective measures might have revealed early functional benefits that were not captured by the mHHS.

### 4.2. The Pain–Function Dissociation: Mechanisms and Clinical Implications

The divergent outcomes for pain and function warrant careful interpretation (detailed stratified analyses are presented in Supplementary Tables S1 and S2). Five mechanisms may explain why ESWT reduced pain but not function:

1. Different Recovery Timelines

Although the anti-inflammatory effects of ESWT can mediate relatively rapid nociceptive relief [19,20,21], the current results demonstrate that these pain-modulating effects are not transient or confined only to the acute phase. In this study, ESWT group patients exhibited a statistically faster and more sustained reduction in pain—measured by the VAS—at 3, 6, and 12 months after nailing compared to controls. This suggests that the benefits of ESWT extend beyond acute pain management, supporting prolonged pain control through the bone union [22,23] and tissue remodeling phases, without adverse effects or interference with fracture healing. The durability of these results highlights ESWT’s potential role in optimizing both early and long-term recovery following hip fracture surgery.

2. Outcome Measure Sensitivity

The mHHS is a validated composite score [24] but may lack sensitivity for detecting early, abductor-specific functional improvements in the immediate postoperative period [25]. Abductor-specific measures (Trendelenburg test, gait analysis, abductor strength dynamometry) might reveal subtle improvements missed by global functional assessment.

3. Ceiling Effects

Functional scores, as assessed by the mHHS at the 3-month time point, were already relatively high (control 57.7 ± 10.5, ESWT 58.7 ± 16.2) despite substantial residual pain in both groups [18,26,27]. This pattern likely reflects the effects of rapid early mobilization protocols following IM nailing, enabling most patients to regain essential ambulatory function within the initial postoperative months. Nevertheless, many patients reported good objective function while still experiencing significant pain, suggesting that pain and functional recovery may follow partially independent trajectories in this population, and highlighting the possibility of nonlinear pain–function relationships after hip fracture surgery.

4. Time-Course Mismatch

Although pain following hip fracture and IM nailing is often assumed to decrease most rapidly in the early postoperative period, our findings demonstrate a distinctive trajectory in which ESWT-treated patients exhibited relatively higher pain scores at initial follow-ups but achieved greater pain reduction over time. The so-called ‘golden cross’ observed between groups, with the ESWT group surpassing controls in late pain improvement, suggests that early application of shockwave therapy may enhance biological healing of both soft tissues and bone, resulting in durable, long-term pain control [12,17,28]. This pattern supports the hypothesis that ESWT not only modulates acute nociceptive pathways but also positively influences the underlying tissue regeneration and bone union process—leading to sustained pain relief extending well beyond the initial months after surgery

5. Mechanistic Mismatch

ESWT primarily targets inflammation and nociceptive pathways, thus providing a biological foundation for enhanced tissue healing and early pain modulation following hip fracture and IM nailing. However, complete functional recovery requires not only resolution of inflammation and pain but also restoration of muscle strength, joint proprioception, and dynamic postural control—mechanisms that are not directly addressed by shockwave therapy alone. In our cohort of elderly patients, these age-related physiological limitations may have inherently restricted the degree of tissue remodeling and neuromuscular adaptation achievable by ESWT. It is plausible that more systematic, progressive resistance training and personalized rehabilitation—when combined with ESWT—would further support muscle hypertrophy, reinnervation, and sensorimotor recovery [29,30], ultimately leading to superior long-term functional outcomes. These findings underscore the need to view ESWT as one critical component of an integrated postoperative rehabilitation program, rather than a stand-alone intervention, especially in older adults with diminished regenerative capacity.

Clinical Implication: These findings suggest ESWT is optimally positioned as a pain-management adjunct that facilitates early rehabilitation engagement, rather than as a comprehensive functional recovery intervention. Combined approaches—ESWT for pain plus structured physical therapy for strength and neuromuscular control—may be necessary to optimize outcomes.

### 4.3. Clinical Implications and Comparison with Literature

From a clinical standpoint, our findings suggest a new potential pathway for enhanced recovery after hip fracture surgery. ESWT can be conceptualized as a “pre-rehabilitation” modality, administered in the first two postoperative weeks. By managing acute pain non-pharmacologically, it may reduce the need for opioid analgesics and their associated side effects, such as delirium and sedation, which are particularly detrimental in this vulnerable elderly population. This could, in turn, make patients more alert and capable of engaging meaningfully with subsequent physical therapy. This approach aligns with the principles of modern perioperative care, which emphasize minimizing opioid use and facilitating early mobilization.

When contextualized with existing literature, our results are broadly consistent. The pain-relieving effects of ESWT in acute injuries have been well-documented in systematic reviews [14,15,16,28,31,32]. Our study extends these findings to a surgically induced injury in a geriatric population, demonstrating its safety even in the presence of metallic implants (with appropriate depth setting). Unlike transient interventions such as femoral nerve blocks, as described by Morrison et al. [33], ESWT appears to offer a more sustained effect on the pain trajectory. Moreover, compared to high-intensity physiotherapy, which requires significant patient effort and motivation as shown by Kimmel et al. [29], ESWT is a passive modality that can be applied even when patients are unable to participate actively in rehabilitation. By reducing pain, ESWT may enable very early ambulation, which is critical for accelerating recovery [34].

### 4.4. Limitations and Future Directions

This study has several important limitations that warrant discussion:

1. Retrospective Trial Registration. The trial was registered retrospectively (CRIS PRE20250926-004) due to administrative delays at enrollment. While this may introduce a reporting bias risk, this is partially mitigated by: (a) the protocol and primary outcomes were established before patient enrollment; (b) outcome assessors were blinded to group allocation; (c) all pre-specified outcomes (VAS, mHHS) were consistently measured at all scheduled time points.
2. Open-Label Design and Placebo Effects. The open-label design and lack of sham control prevent definitively ruling out placebo effects. This is a critical limitation for ESWT evaluation. Future double-blind, sham-controlled trials using matched transducers are necessary to isolate ESWT-specific effects from psychological and contextual factors.
3. Limited Sample Size and Statistical Power. With *n* = 51 (25 control, 26 ESWT), statistical power for subgroup analyses is limited. Findings from stratified analysis—particularly for right-sided fractures (ESWT *n* = 4)—should be interpreted with appropriate caution due to the very small subsample size. Large-scale multicenter trials are needed to confirm efficacy, evaluate generalizability across populations, and provide sufficient power for clinically meaningful subgroup analyses.
4. Single-Center Design and Generalizability. Conduct at a single tertiary academic medical center in Korea may limit generalizability. Results may not reflect outcomes in community hospitals, different healthcare systems, different cultural contexts, or populations with different demographic characteristics or comorbidity profiles.
5. Baseline Imbalance in Fracture Laterality (NOW ADDRESSED). A notable baseline imbalance in fracture laterality was observed between groups (left-sided: 40.0% in control vs. 86.2% in ESWT; χ^2^ = 8.756, *p* = 0.003). However, rather than representing an unresolved confounding problem, this was comprehensively addressed through: (a) sensitivity analysis including laterality as a fixed covariate in the mixed-effects model (Supplementary Table S1), with results unchanged; and (b) stratified analysis for left-sided and right-sided fractures separately (Supplementary Table S2). These additional analyses demonstrated that primary findings were robust to the baseline imbalance, suggesting it did not confound treatment effects.
6. Unmeasured Confounding Variables. We did not systematically assess psychological status, patient motivation, family support, socioeconomic factors, or cognitive status—all of which influence postoperative recovery and rehabilitation engagement. Future comprehensive studies should incorporate validated instruments for these psychosocial and economic dimensions.
7. Limited Sensitivity of Functional Outcome Measure. While the mHHS is psychometrically sound, it may lack sensitivity for detecting early, abductor-specific functional improvements in the immediate postoperative period. Performance-based measures (gait speed, Trendelenburg test, abductor strength via dynamometry) might reveal functional benefits not captured by this composite score.
8. Absence of Economic Analysis. This study did not include formal cost-effectiveness or economic analysis. ESWT equipment acquisition costs, maintenance, and personnel time must be weighed against potential savings from reduced analgesic consumption, shorter rehabilitation duration, and earlier return to work or activity. Comprehensive health economic evaluation is essential before clinical implementation and resource allocation decisions can be confidently recommended.

## 5. Conclusions

In this open-label randomized controlled trial, early ESWT demonstrated statistically significant improvement in pain trajectories following IM nailing for hip fractures, with effects that remained robust after adjusting for baseline imbalances. While the magnitude of benefit was modest (approximately 1.0-point VAS reduction over 12 months) and functional improvements did not reach statistical significance, ESWT could represent a safe adjunctive option for postoperative pain management. The clinical significance of these findings, optimal patient selection criteria, and cost-effectiveness require further investigation in larger, well-controlled trials before widespread implementation can be recommended.

## Figures and Tables

**Table 1:** Baseline Demographics and Clinical Characteristics of Patients.

Characteristic		ESWT		*p*-Value
		Control (*n* = 25)	ESWT (*n* = 26)	
Age		79.84 ± 7.88	81.19 ± 8.86	0.567
Sex (%)	Female	17 (68.0)	23 (88.5)	0.151
	Male	8 (32.0)	3 (11.5)	
Site (%)	Lt	10 (40.0)	22 (84.6)	0.003
	Rt	15 (60.0)	4 (15.4)	
Diagnosis (%)	femur neck stress fracture	0 (0.0)	1 (3.8)	1.000
	intertrochanteric fracture	20 (80.0)	21 (80.8)	
	midshaft fracture	2 (8.0)	2 (7.7)	
	midshaft peri-implant fracture	1 (4.0)	0 (0.0)	
	proximal shaft fracture	1 (4.0)	0 (0.0)	
	subtrochanteric fracture	1 (4.0)	2 (7.7)	
Preop.Koval.Gr (%)	1	15 (60.0)	11 (42.3)	0.397
	2	5 (20.0)	3 (11.5)	
	3	1 (4.0)	5 (19.2)	
	4	1 (4.0)	2 (7.7)	
	5	3 (12.0)	4 (15.4)	
	6	0 (0.0)	1 (3.8)	

Note: Group sizes are consistently ESWT (*n* = 26) and Control (*n* = 25) across all rows.

**Table 2:** Comparison of Clinical Outcomes (VAS and mHHS) at Each Follow-up Point.

	Control	ESWT
*n*	25	26
Baseline VAS	6.08 ± 1.59	6.31 ± 1.74
3-month VAS	3.68 ± 1.55	3.46 ± 1.83
6-month VAS	2.72 ± 1.73	2.16 ± 1.87
12-month VAS	1.72 ± 1.80	0.89 ± 1.47
3-month mHHS	57.72 ± 10.52	58.65 ± 16.20
6-month mHHS	62.72 ± 11.06	64.38 ± 16.68
12-month mHHS	67.72 ± 11.06	73.27 ± 14.35
VAS_diff(base-3 m)	1.86 ± 1.98	2.04 ± 2.60
VAS_diff(base-6 m)	2.62 ± 2.25	3.39 ± 2.79
VAS_diff(base-12 m)	3.57 ± 2.29	4.35 ± 2.08

**Table 3:** Regression Results: VAS.

Characteristic	Beta	95% CI	*p*-Value
	0.337	0.289–0.392	<0.001
Month (per month)			
Group (ESWT vs. Control, ref = Control)	—	—	
	0.125	−0.659–0.908	*p* = 0.755
Month × ESWT (interaction)	0.086	0.010–0.162	*p* = 0.027

Model specification: VAS ~ Month + Group + Month × Group (linear regression). Month was entered as an integer (months from baseline). Group was coded 1 = ESWT and 0 = Control (reference). The symbol “×” denotes the interaction term between Month and Group. Beta indicates the estimated change in VAS per unit increase in Month or relative to the reference level of Group. CI = confidence interval.

**Table 4:** Regression Results: mHHS.

Characteristic	Beta	95% CI	*p*-Value
Month (per month)	4.732	3.810–5.655	<0.001
Group (ESWT vs. Control, ref = Control)	—	—	
	−0.451	−9.421, 8.519	*p* = 0.9
Month × ESWT (interaction)			
	0.485	−0.807, 1.777	0.462

Model specification: mHHS ~ Month + Group + Month × Group (linear regression). Month was entered as an integer (months from baseline). Group coded as 1 = ESWT and 0 = Control (reference). “×” denotes the interaction term between Month and ESWT group. Beta = estimated change in mHHS per unit increase (Month) or relative to reference (Group). CI = confidence interval.

**Table 5:** Responders with ≥8-point mHHS Increase.

Interval	Control (*n* = 25)	ESWT (*n* = 26)
6 m–3 m	7/25 (28.0 %)	3/26 (11.5 %)
12 m–6 m	9/25 (36.0 %)	7/26 (26.9 %)
12 m–3 m	16/25 (64.0 %)	13/26 (50.0 %)

## Data Availability

The data presented in this study are available on request from the corresponding author. The data are not publicly available due to ethical and privacy restrictions.

## Supplementary Materials

The following supporting information can be downloaded at: https://www.mdpi.com/article/10.3390/life15111704/s1, Table S1: Sensitivity Analysis Results; Table S2: Stratified Analysis (by Laterality).
