# Supplementary material for: Effect of Early Extracorporeal Shockwave Therapy on Postoperative Pain and Functional Recovery After Intramedullary Nailing: An Open-Label Randomized Controlled Trial
Source: Life (Basel). 2025 Nov 3;15(11):1704. doi: 10.3390/life15111704 (PMC12653659; doi:10.3390/life15111704)
Supplement: Supplementary file 1 [file life-15-01704-s001.zip › Table S1 Sensitivity Analysis Results.pdf]

Table 1: Mixed-Effects Model Results for VAS with Laterality Adjustment

Characteristic	Beta	95% CI	p-value
Model 1: Unadjusted			
ESWT 3 months	0.142	-0.508, 0.791	0.669
ESWT 6 months	-0.348	-0.841, 0.146	0.168
ESWT 12 months	-0.617	-1.111, -0.123	0.014
Model 2: Adjusted for Laterality			
ESWT 3 months	-0.079	-0.809, 0.650	0.831
ESWT 6 months	-0.223	-0.781, 0.336	0.435
ESWT 12 months	-0.631	-1.189, -0.072	0.027
Laterality main effect (3 months)	-0.495	-1.249, 0.259	0.198
Laterality × Month 6 interaction	0.280	-0.297, 0.858	0.341
Laterality × Month 12 interaction	-0.031	-0.608, 0.546	0.916
