# Supplementary material for: Effect of Early Extracorporeal Shockwave Therapy on Postoperative Pain and Functional Recovery After Intramedullary Nailing: An Open-Label Randomized Controlled Trial
Source: Life (Basel). 2025 Nov 3;15(11):1704. doi: 10.3390/life15111704 (PMC12653659; doi:10.3390/life15111704)
Supplement: Supplementary file 1 [file life-15-01704-s001.zip › Table S2 Stratified Analysis (by Laterality).pdf]

Table 2: Stratified Analysis of VAS Outcomes by Fracture Laterality

Characteristic	Beta	SE	95% CI	p-value
Left-sided Fractures (n=32: Control=10, ESWT=22):				
VAS Month 3	-0.200	0.362	-0.909, 0.509	0.680
VAS Month 6	-0.173	0.418	-0.992, 0.646	0.734
VAS Month 12	-0.745	0.374	-1.479, -0.012	0.056
Right-sided Fractures (n=19: Control=15, ESWT=4):				
VAS Month 3	0.183	0.645	-1.081, 1.448	0.814
VAS Month 6	-0.583	0.542	-1.645, 0.478	0.447
VAS Month 12	-0.633	0.461	-1.538, 0.271	0.135
